# Problematic Smartphone Use, Deep and Surface Approaches to Learning, and Social Media Use in Lectures †

**DOI:** 10.3390/ijerph15010092

**Published:** 2018-01-08

**Authors:** Dmitri Rozgonjuk, Kristiina Saal, Karin Täht

**Affiliations:** 1 Institute of Psychology, University of Tartu, Tartu 50409, Estonia; kristiinasaal@gmail.com (K.S.); karin.taht@ut.ee (K.T.); 2 Department of Psychology, University of Toledo, Toledo, OH 43606, USA

**Keywords:** problematic smartphone use, smartphone addiction, social media, approaches to learning, deep approach to learning, surface approach to learning

## Abstract

Several studies have shown that problematic smartphone use (PSU) is related to detrimental outcomes, such as worse psychological well-being, higher cognitive distraction, and poorer academic outcomes. In addition, many studies have shown that PSU is strongly related to social media use. Despite this, the relationships between PSU, as well as the frequency of social media use in lectures, and different approaches to learning have not been previously studied. In our study, we hypothesized that both PSU and the frequency of social media use in lectures are negatively correlated with a deep approach to learning (defined as learning for understanding) and positively correlated with a surface approach to learning (defined as superficial learning). The study participants were 415 Estonian university students aged 19–46 years (78.8% females; age M = 23.37, SD = 4.19); the effective sample comprised 405 participants aged 19–46 years (79.0% females; age M = 23.33, SD = 4.21). In addition to basic socio-demographics, participants were asked about the frequency of their social media use in lectures, and they filled out the Estonian Smartphone Addiction Proneness Scale and the Estonian Revised Study Process Questionnaire. Bivariate correlation analysis showed that PSU and the frequency of social media use in lectures were negatively correlated with a deep approach to learning and positively correlated with a surface approach to learning. Mediation analysis showed that social media use in lectures completely mediates the relationship between PSU and approaches to learning. These results indicate that the frequency of social media use in lectures might explain the relationships between poorer academic outcomes and PSU.

## 1. Introduction

Since their introduction in 2009, smartphones have had a significant impact on daily life across the world. The worldwide ownership of smartphones is around 43% [1], and more than 50% of Estonians own a smartphone [2]. Smartphones have several advantages in educational settings, allowing one to take notes, browse for information, communicate with others, and use specific applications for learning skills [3]. Several studies have shown, however, that there are instances where the excessive use of smartphones leads to the development of “problematic smartphone use” (PSU), a phenomenon characterized by the occurrence of addictive-like symptoms [4]. PSU has been associated with psychopathological symptoms [5] as well as poor academic outcomes [6,7]. Similar findings have been reported with excessive social media use [8]. The aim of this paper is to investigate how PSU is related to approaches to learning. Specifically, this study addresses the questions of how PSU is related to learning for understanding (known as the deep approach to learning) and learning motivated by external incentives (for example, grades; known as a surface approach to learning) [9]. In addition, the mediating effect of social media use in lectures is analyzed, as it may be the case that the relationship between PSU and approaches to learning is dependent on the frequency of social media use.

Previous works regarding smartphone addiction is often seen as controversial. It has been debated whether or not smartphone “addiction” is a behavioral addiction, and whether or not it may be considered as an actual addiction from the perspective of contemporary addiction theories [10,11,12]. Nevertheless, one cannot neglect the growing body of evidence that suggests that problematic smartphone usage is related to several psychopathological symptoms, such as depression and anxiety [5,13], poorer health and sleep quality [14], and lower academic achievements [6]. In addition to smartphone addiction, the following terms have been used to describe conceptually the same phenomenon: proneness to smartphone addiction [15,16], smartphone overuse [17,18], excessive smartphone use [19], problematic mobile phone use [20], mobile phone dependence [21], and mobile phone addiction [22,23]. In essence, these terms characterize the excessive use of smartphones accompanied by symptoms resembling those found in contemporary addiction models: dependence, withdrawal, tolerance, and functional impairment [4]; therefore, we conceptualize the phenomenon as problematic smartphone use.

PSU as a maladaptive coping method [24] has received relatively little attention in academic contexts in scientific literature. Although studies have found that the higher levels of PSU are related to poor academic outcomes [6,7], different approaches to learning as potential causal factors for academic outcomes have not yet been researched in the light of problematic technology use.

Empirical findings have led Marton and Säljö [9] to propose a distinction between approaches to learning; these were categorized as surface and deep approaches to learning. Accordingly, one uses a deep approach to learning to fully understand the content studied. In contrast, the surface approach to learning is characterized by instrumental learning which aims to minimally fulfill the requirements of study; with the latter, only the basics of the study material are learned [25]. Biggs [26] defined both of these approaches as motives and strategies. While a deep motive involves intrinsic interest in the content that is learned, and the aim is to develop competence, a surface motive is to meet requirements minimally. In addition, deep strategy is to discover meaning by reading widely, relating one’s previous knowledge with learned material, whereas surface strategy is to limit the amount of work one needs to do (through rote learning).

It has been shown that a deep approach to learning facilitates critical thinking, finding causal relationships, creativity, and autonomous thinking. Therefore, this type of approach is an essential ingredient in successful learning, as it leads to a more systematic and thorough comprehension of information [27]. Authors [28] have found that those with a deep approach to learning have better developed organization skills, more spontaneously expressed ideas, and clearer, longer, and more detailed explanations. Those with a deep approach to learning may also make more associations with their work and prior knowledge and experiences [28]. Other works have linked deep learning with a higher need to understand material and a drive to satisfy one’s curiosity [29,30]. On the other hand, a surface approach to learning is characterized by incentive-oriented strategy, according to which the student aims to do only as much as is necessary to study, often compulsory, material [25,29]. Those who are prone to this approach tend to focus on learning facts, isolated details, examples and illustrations without connecting the pieces of information to their knowledge to provide a broader perspective [30]. This leads to a focus on understanding simpler principles [27]. Surface learners are rather passive and their attitude towards learning is to regard it as something which inevitably happens. In comparison, a deep approach to learning leads to an increased sense of control over the learning process [9].

As the information suggests, relationships between academic success and learning strategies are well researched. Several studies have shown that surface learners have lower academic outcomes and are less successful in school, whereas those with a deep approach to learning are more successful in school [31,32,33,34,35]. Additionally, the probability of graduating from higher education institutions is lower for students who study superficially [36].

In addition, it has been found that more social media use (on the example of Facebook) is related to worse academic outcomes [37]; also, engaging with social distractors (social media with its functionalities could be considered as one) have shown to be related to poorer concentration and procrastination [38]. Unsurprisingly, one of the main functions of smartphones is social media use [39,40,41,42], allowing for almost constant access to continually changing content and engaging with social distractors.

Though some studies have shown that higher levels of PSU and other excessive technology use (e.g., the Internet) are associated with poorer academic outcomes [6,7,43,44], it has not been studied if different levels of PSU are related to deep and surface approach to learning. Furthermore, the role of social media use in lectures is not investigated in the potential relationships between the levels of PSU, and deep and surface approaches to learning.

The aims of this study are (a) to investigate how deep and surface approaches to learning are related to the levels of PSU and social media use in lectures, and (b) to clarify if social media use in lectures mediates the relationship between the levels of PSU and approaches to learning. Based on the empirical findings discussed earlier, we have posed the following hypotheses:

**Hypothesis** **1 (H1).** The levels of PSU are negatively related to a deep approach to learning and positively related with a surface approach to learning. PSU is a maladaptive coping method [24] that has been found to be associated with several other dysfunctional behaviors or detrimental outcomes, including in academic settings [6,7]. Therefore, we expect the levels of PSU to be correlated negatively to the deep approach to learning (adaptive learning strategy) and positively to the surface approach to learning (maladaptive learning strategy).

**Hypothesis** **2 (H2).** The frequency of social media use in lectures is negatively correlated to a deep approach to learning and positively related to a surface approach to learning. Studies have shown that more social media use is related to poorer academic outcomes [37]. Similarly to the previous hypothesis, we expect social media use in lectures to be related to approaches to learning.

**Hypothesis** **3 (H3).** The levels of PSU are positively correlated with the frequency of social media use in lectures. The levels of PSU have been shown to be related to the social use of smartphones [39,40,41,42]. We expect that is also the case with social media use in lectures, specifically.

**Hypothesis** **4 (H4).** Social media use in lectures mediates the relationship between the levels of PSU and approaches to learning. It has been found that levels of PSU are related to social media use and social use in general [15,42,45]. It has also been shown that more social media use is associated with lower academic outcomes [37]. We hypothesize that higher levels of PSU is related to more social media use in lectures, and this is related with a less deep and more surface approach to learning.

## 2. Materials and Methods

### 2.1. Sample and Procedure

Four hundred fifteen Estonian university students (ages ranging from 19 to 46 years, M_age_ = 23.37 ± 4.19 years; 78.8% were female) participated in an online survey in Estonian. Ten participants had missing data in their responses and were, therefore, removed from further analyses. The characteristics of the effective sample are the following: ages ranging from 19 to 46 years, M_age_ = 23.33 ± 4.21 years; 79.0% were female. Therefore, the efficient sample comprised 405 participants (age range from 19 to 46 years, M_age_ = 23.33 ± 4.21 years; 79.0% were female). Three hundred ninety-three (97.04%) participants marked their mother tongue as Estonian, 11 (2.7%) as Russian, and 1 as “other.”

The participants were recruited through university mailing lists and via social media. Prior to taking the survey, participants filled out an informed consent form in accordance with the Declaration of Helsinki. The participants were asked to complete a merged questionnaire that consisted of the following parts: (a) general socio-demographics, (b) the Estonian Revised Study Process Questionnaire, (c) questions about social media use in lectures, (d) the Estonian Smartphone Addiction Proneness Scale.

### 2.2. Explanations of Measures

Further details of the measures used are provided.

#### 2.2.1. General Socio-Demographics

These included items about the participants’ age (in full years), gender (male/female/other), and mother tongue (Estonian/Russian/other).

#### 2.2.2. The Revised Study Process Questionnaire (Estonian Adaptation)

(R-SPQ-2F; [46]): this is a modified two-dimensional 16-item questionnaire with Likert-type scale (from 1 = “do not agree at all” to 5 = “totally agree”) that is inspired by the Revised Study Process Questionnaire developed by Biggs, Kember, and Leung [25]; see [46] for further details about the adaptation process and Table S1 for the items of the scale. The scale measures the extent to which students use a surface (e.g., “I generally restrict my study to what is specifically set as I think it is unnecessary to do anything extra”) or deep (e.g., “I find that at times studying gives me a feeling of deep personal satisfaction”) approach to learning as their learning strategy. The internal reliability was acceptable for the surface approach to learning subscale (Cronbach’s α = 0.72), and for the deep approach to learning subscale (Cronbach’s α = 0.75).

#### 2.2.3. Social Media Use in Lectures

Participants were asked two questions about social media use in lectures, derived from the items and scales used in [47]: firstly, “How frequently do you keep track of social media in lectures?” (1 = “never” … 5 = “all the time”) and, secondly, “How often do you communicate with friends using social networking sites (Facebook, Twitter, etc.) in lectures?” (1 = “never” … 5 = “all the time”). These items were highly correlated (r = 0.74, *p* < 0.0001) and were aggregated into one social media use index.

#### 2.2.4. The Estonian Smartphone Addiction Proneness Scale

(E-SAPS18; [15]: The E-SAPS18 is a five-dimensional questionnaire consisting of 18 items measuring the frequency of smartphone addiction symptoms on a 6-point Likert scale (from 1 = “strongly disagree” to 6 = “strongly agree”). E-SAPS18 consists of five factors (tolerance, positive anticipation, cyberspace-oriented relationships, withdrawal, and physical symptoms), with a higher-order factor of smartphone addiction proneness. The internal reliability of E-SAPS18 is very good (Cronbach’s α = 0.87), and E-SAPS18 has been validated against other instruments measuring Internet and smartphone addiction proneness.

### 2.3. Data Analysis

RStudio version 3.2.3 (R Core Team, Vienna, Austria) was used for descriptive statistics and correlation analysis. The skewness and kurtosis of distributions of variables were among the acceptable ranges (from −2 to +2) [48,49]. One-tailed Pearson product–moment correlation was used to analyze bivariate relationships between variables, as we expected directional relationships in each correlation. Mplus version 8 was used for mediation analysis.

We used the weighted least squares estimation with a mean- and variance-adjusted chi-square (WLSMV), because it has been shown to be less biased and more accurate than similar estimation methods (e.g., robust maximum likelihood, or MLR) for the data type [50]. Using the WLSMV estimation method, we treated the items of social media use in lectures and E-SAPS18 as categorical/ordinal data, thus involving a polychoric covariance matrix and probit regression coefficients [51]. The summed scale scores for deep and surface approach to learning were treated as observed variables in the models. The cross-products of direct effects to compute mediation effect were used, applying the Delta method for computing indirect effect standard errors, with non-parametric bootstrapping across 1000 samples [52]. Goodness of fit was judged by standard parameters: (a) Comparative Fit Index (CFI) ≥ 0.90, (b) Tucker–Lewis Index (TLI) ≥ 0.90, and (c) root mean square error of approximation (RMSEA) ≤ 0.08.

We used SPSS version 24 to compute Mahalanobis distances to detect the outliers in the multivariate dataset. Altogether, there were 19 cases (4.7%) where *p* < 0.05 (4 cases, or 1% of the sample, with *p* < 0.01). The analyses described above were re-ran in order to detect if these statistical outliers influenced the results cardinally. However, as the results were virtually the same (signs and magnitudes of coefficients, statistical significance of the predictors, and the mediating variable), we retained these multivariate outliers in the sample.

## 3. Results

### 3.1. Correlation Analysis

Summary and scale reliability statistics for the study sample are highlighted in Table 1. In order to analyze the relationships between PSU, approaches to learning, and social media use in lectures, Pearson product–moment correlation analyses were carried out. The results are shown in Table 1.

It could be observed that all variables are statistically significantly inter-related. Specifically, PSU (as an E-SAPS18 score) is in positive correlation with social media use in lectures and a surface approach to learning; the latter two variables are, in turn, also positively correlated with each other. Additionally, both PSU and social media use in lectures are negatively correlated with a deep approach to learning.

### 3.2. Mediation Analysis

Confirmatory factor analysis showed that the five-factor E-SAPS18 with one higher-order factor had an acceptable fit: CFI = 0.93; TLI = 0.92; RMSEA = 0.09. The fit indices for two-factor R-SPQ-2F (Estonian adaptation) were CFI = 0.76, TLI = 0.72, and RMSEA = 0.13. Because the latter scale did not show a great fit, we used observed variables (summed scores for deep and surface approach to learning) as outcome variables.

Three mediation models were constructed to investigate if social media use in lectures mediates the relationship between PSU and approaches to learning. In the first model (Model 1), we used only the deep approach to learning as the outcome variable. In the second model (Model 2), we used the surface approach to learning as the outcome model. In both of these models, the mediating variable was controlled for age and gender, as it has been shown that age and gender might impact social media engagement [53]. The third model (Model 3) included both the deep and surface approaches to learning as outcome variables predicted by the levels of PSU and mediated by social media use in lectures. Again, the mediator was controlled for gender; furthermore, deep and surface approaches to learning were specified to covary in that model. As mentioned earlier, measures of PSU and social media use in lectures were modeled as latent (categorical/ordinal), and the summed scores for deep and surface approaches to learning and the covariates (age, gender) were treated as observed variables.

The fit indices for Model 1 were CFI = 0.94, TLI = 0.93, and RMSEA = 0.07; for Model 2, they were CFI = 0.94, TLI = 0.93, and RMSEA = 0.07; and for Model 3: CFI = 0.94, TLI = 0.93, and RMSEA = 0.07. The coefficients of the models are presented in Table 2.

It could be observed from all three models that social media use in lectures mediates the relationship between problematic smartphone use and approaches to learning. In Model 1, PSU does not have a direct effect on deep approach to learning; however, social media use in lectures completely mediates the relationship between PSU and deep approach to learning. Furthermore, the indirect effect is negative.

Similarly, social media use in lectures completely mediates the relationship between PSU and surface approach to learning (see Model 2). However, in this case, the indirect effect is positive. Higher PSU leads to higher social media use in lectures which, in turn, is associated with more surface approach to learning. 

These effects are present even if both surface and deep approach to learning are included in the same model as outcome variables. Interestingly, gender is not associated with social media use in lectures, whereas in all models, younger people tend to use more social media in lectures.

## 4. Discussion

According to previous research, PSU and excessive social media use relate to poorer academic outcomes [6]. However, these relationships are often examined in relation to grades or grade point average (GPA), not to deep and surface approaches to learning. As such, in our study, we hypothesized that PSU would be negatively related to a deep approach to learning and positively related to a surface approach to learning. Indeed, the results showed that a higher PSU is associated with less commitment to a deep approach to learning and with greater commitment to a surface approach to learning. We expected, too, that more frequent social media use in lectures would be negatively correlated with a deep approach to learning and positively associated with a surface approach to learning. Our results also confirmed this hypothesis. The relationship between frequent, engaged smartphone use and multitasking [54,55], which may be detrimental to academic achievement [56], may potentially explain these findings. Those with additional access to technology may be more prone to multitasking [57], which may in turn lead to less in-depth or more superficial learning. Future studies should try to take into account multitasking as a potential factor in influencing the relationships reported in this study.

According to our third hypothesis, we expected PSU to be positively associated with the frequency of social media use in lectures. This hypothesis was confirmed by the results. This result is not surprising, as one of the most popular activities on smartphones is social media usage. Therefore, it is natural that those with higher PSU are also more prone to use social media in lectures.

As a second aim of the study, we presented a conceptual mediation model where the relationship between PSU and approaches to learning are mediated by the frequency of social media use in lectures. Our previous results provided the statistical basis for the models [58]. According to the models, social media use in lectures completely mediated the relationship between PSU and approaches to learning. In other words, though PSU and approaches to learning were related in bivariate correlations, these relationships could be explained by the effect of social media use frequency. Indeed, it has been indicated that the most frequent activity carried out on smartphones is social media use [15,45]. Smartphones allow for mobility and availability in terms of online interactions and content consumption from social media platforms.

Several limitations are worth mentioning. Firstly, self-report measures were used, and this might affect the reliability and ecological validity of the findings. It would be interesting to contrast our results with behavioral social media usage data (e.g., the actual measured time of social media usage) and objective academic data (e.g., GPA, test scores, and SAT scores). This kind of approach would allow for more accurate and reliable data, and better generalization. Secondly, the study design was cross-sectional; therefore, one has to be cautious in making causal interpretations. This is especially important to keep in mind while interpreting the results of mediation models. Our hypothesized models are based on the logic that PSU, as a more generic construct, encompasses social media use and that it therefore could be a precondition for more social media consumption. However, it could also be the case that more social media use causes more PSU. Additionally, students’ approaches to learning could be influenced by both PSU and social media use; however, it could also be the case that approaches to learning impact how much social media a student consumes in lectures. Finally, we did not ask the participants how much time they spent online (per day or per week) and why the participants engaged in activities involving social media use in lectures. Though studies have shown that self-reported estimated time of technology use is typically inaccurate in comparison to objective use [59,60,61], knowing learning about the objective usage time and could greatly improve the understanding of these results. Additionally, distinguishing between the activities in which students engage on social media (e.g., learning vs. entertainment that is not related to class content) could specify the potential effects. We encourage the reader to be mindful of these limitations, and we consider experimental and/or longitudinal studies to be helpful in clarifying this issue; we hope the current paper inspires studies of that nature.

This study has both theoretical and practical implications. From a theoretical perspective, this is the first study to investigate the relationships between approaches to learning and PSU. Though it has been shown that a deep approach to learning is positively related to academic outcomes, and a surface approach to learning is found to have negative effects on academic outcomes [31,32,33,34], this study provides potential evidence to the findings where PSU has been associated with poor academic outcomes [6]. Specifically, poorer outcomes might be due to a less deep and/or more surface approach to learning. In addition, the findings between PSU and academic outcomes could be explained by students engaging in more social media use.

Among the potential practical implications, this paper suggests that social media use in lectures might be the driver in the relationship between approaches to learning and PSU. Lecturers might consider restricting access to social media use in lectures or to advise their students that engaging in excessive smartphone use and especially social media use in lectures could have detrimental effects on their academic outcomes. However, this idea needs further testing—preferably by implementing experimental and, if possible, longitudinal study design to better understand if this practical suggestion derived from the results of this study have beneficial effects.

## 5. Conclusions

Recent advances in technology offer many promising advantages in the classroom. However, problematic technology use has been shown to be negatively related to academic outcomes. In the current study, we found that both PSU and social media use in lectures are related to less commitment to a deep approach to learning (learning to fully understand the content studied) in place of a more surface approach to learning (instrumental learning to meet the requirements of one’s learning outcomes). Additionally, we proposed hypothetical models where the frequency of social media use in lectures mediates the relationship between PSU and approaches to learning. We found that social media use in lectures completely mediates the aforementioned relationship. Further studies should address the limitations of this study, such as using self-reported measures and cross-sectional study design.

## Figures and Tables

**Table 1:** Pearson product–moment correlations, means, standard deviations, and internal consistencies of scales and subscales.

Variable		1	2	3	Min	Max	M	SD	α
1	PSU				18	76	35.52	10.73	0.86
2	SMUL	0.326 ***			2	10	5.92	2.10	0.85
3	DA	−0.109 *	−0.304 ***		11	39	26.80	4.62	0.78
4	SA	0.177 ***	0.297 ***	−0.425 ***	8	34	20.12	4.93	0.75

Notes: *N* = 415. PSU = problematic smartphone use measured by the E-SAPS18; SMUL = Social media use in lectures; DA = deep approach to learning; SA = surface approach to learning. * *p* ≤ 0.05, *** *p* ≤ 0.001.

**Table 2:** The results of mediation models.

**Model 1 (Outcome DA)**				
**Covariates**	**Bivariate B (SE)**	***t***	**Multivariate B (SE)**	***t***
PSU	0.001 (0.079)	0.016	0.001 (0.061)	0.016
SMUL	−0.345 (0.060)	−5.751 ***	−0.417 (0.063)	−6.630 ***
PSU -> SMUL	0.547 (0.098)	5.573 ***	0.348 (0.059)	5.874 ***
Age -> SMUL	−0.095 (0.012)	−7.799 ***	−0.440 (0.053)	−8.349 ***
Gender -> SMUL	0.103 (0.139)	0.744	0.046 (0.061)	0.754
PSU -> SMUL -> DA	−0.189 (0.050)	−3.757 ***	−0.145 (0.035)	−4.115 ***
**Model 2 (Outcome SA)**				
**Covariates**	**Bivariate B (SE)**	***t***	**Multivariate B (SE)**	***t***
PSU	0.088 (0.061)	1.457	0.093 (0.061)	1.513
SMUL	0.205 (0.050)	4.136 ***	0.336 (0.076)	4.410 ***
PSU -> SMUL	0.548 (0.094)	5.820 ***	0.350 (0.056)	6.247 ***
Age -> SMUL	−0.082 (0.011)	−7.369 ***	−0.382 (0.047)	−8.055 ***
Gender -> SMUL	0.103 (0.124)	0.829	0.046 (0.055)	0.839
PSU -> SMUL -> SA	0.112 (0.035)	3.193 ***	0.118 (0.035)	3.340 ***
**Model 3 (Outcomes DA and SA)**				
**Covariates**	**Bivariate B (SE)**	***t***	**Multivariate B (SE)**	***t***
PSU -> DA	0.002 (0.084)	0.024	0.001 (0.060)	0.024
SMUL -> DA	−0.361 (0.060)	−6.008 ***	−0.419 (0.064)	−6.604 ***
PSU -> SA	0.112 (0.071)	1.572	0.097 (0.061)	1.594
SMUL -> SA	0.243 (0.057)	4.261 ***	0.336 (0.074)	4.569 ***
PSU -> SMUL	0.562 (0.102)	5.526 ***	0.353 (0.060)	5.848 ***
Age -> SMUL	−0.097 (0.013)	−7.609 ***	−0.447 (0.053)	−8.364 ***
Gender -> SMUL	0.053 (0.146)	0.365	0.024 (0.064)	0.370
PSU -> SMUL -> DA	−0.203 (0.055)	−3.668 ***	−0.148 (0.037)	−3.969 ***
PSU -> SMUL -> SA	0.137 (0.044)	3.095 **	0.119 (0.037)	3.222 ***
DA <-> SA	−0.228 (0.037)	−6.094 ***	−0.526 (0.051)	−10.365 ***

Notes *N* = 415. PSU = problematic smartphone use measured by the E-SAPS18; SMUL = Social media use in lectures; DA = deep approach to learning; SA = surface approach to learning. ** *p* ≤ 0.01, *** *p* ≤ 0.001.

## Supplementary Materials

The following are available online at www.mdpi.com/1660-4601/15/1/92/s1, Table S1: The 16-Item Revised Study Process Questionnaire (Estonian Adaptation).
