# Supplementary material for: Problematic Smartphone Use, Deep and Surface Approaches to Learning, and Social Media Use in Lectures
Source: Int J Environ Res Public Health. 2018 Jan 8;15(1):92. doi: 10.3390/ijerph15010092 (PMC5800191; doi:10.3390/ijerph15010092)
Supplement: Supplementary file 1 [file ijerph-15-00092-s001.docx]

**Table S1**. the 16-item Revised Study Process Questionnaire (Estonian adaptation).

| **Item in Estonian [in English]** | **Construct** |
| --- | --- |
| 1. *Õppimine pakub mulle sügavat isiklikku rahuldust.* [I find that at times studying gives me a feeling of deep personal satisfaction.] | Deep |
| 2. *Ma õpin tõsiselt ainult seda, mida kästakse.* [I only study seriously what’s given out in class or in the course outlines.] | Surface |
| 3. *Enamik uusi teemasid on minu jaoks huvitavad ja ma olen valmis pühendama lisaaega selleks, et hankida nende kohta lisainfot.* [I find most new topics interesting and often spend extra time trying to obtain more information about them.] | Deep |
| 4. *Kordan materjali, kuni tean seda peast, isegi kui ma sellest aru ei saa.* [I learn some things by rote, going over and over them until I know them by heart even if I do not understand them.] | Surface |
| 5. *Piirdun täpselt antud ülesandega ja arvan, et pole vaja teha midagi enamat.* [I generally restrict my study to what is specifically set as I think it is unnecessary to do anything extra.] | Surface |
| 6. *Õpin hoolega, kui materjal on huvitav.* [I work hard at my studies because I find the material interesting] | Deep |
| 7. *Kulutan palju vaba aega, et leida rohkem infot kursusel käsitletud huvitavate teemade kohta*. [I spend a lot of my free time finding out more about interesting topics which have been discussed in different classes.] | Deep |
| 8. *Teemasid pole mõtet õppida sügavuti, see raiskab ainult aega ja tekitab segadust.* [I find it is not helpful to study topics in depth. It confuses and wastes time, when all you need is a passing acquaintance with topics.] | Surface |
| 9. *Vaatan üle kogu lisamaterjali, mida kursusel soovitatakse. [*I make a point of looking at most of the suggested readings that go with the lectures.] | Deep |
| 10. *Pole mingit mõtet vaadata materjali, mida eksamil tõenäoliselt ei küsita.* [I see no point in learning material which is not likely to be in the examination.] | Surface |
| 11*. Parim tee eksamil läbi saamiseks on õppida pähe vastused küsimustele, mida tõenäoliselt esitatakse.* [I find the best way to pass examinations is to try to remember answers to likely questions.] | Surface |
| 12. *Õpin, et kogeda midagi uut*. [I learn to experience something new.] | Deep |
| 13. *Õpin, kuna tahan maailmast aru saada.* [I learn because I want to understand the world.] | Deep |
| 14. *Antud kodutööd teen ära esimesel võimalusel.* [I do homework on a first occasion.] | Deep |
| 15. *Kui ma aru ei saa, siis õpin pähe.* [If I do not understand some topics I learn them by rote.] | Surface |
| 16. *Raskete teemade puhul on pähe õppimine üks viis eksamil läbi saada.* [In case of difficult topics, learning by rote is one way to pass an exam.] | Surface |
